# Choose Health or Sickness

**Published:** 1883-12

**Authors:** 


### CHOOSE HEALTH OR SICKNESS.

Those who desire and appreciate health should be as willing to make some effort to secure it as they do to obtain the other and good things which increase the pleasures of life. Pure water is essentially necessary to good health. All wells, cisterns and springs should be thoroughly cleaned in the early Spring or in the Autumn. The usual method of placing a large stone on the top of the cistern is injurious to the water unless an aperture is left in the stone and fitted with a wooden cover. The air should not be wholly excluded from the cistern, else moldy conditions will predominate, although perhaps not apparent, and the water will not be wholesome, and in it sometimes there may be found various kinds of insects and reptiles.

Water is the natural drink of all living creatures and it serves several important purposes in the animal economy. Firstly, it repairs the loss of the aqueous part of the blood caused by evaporation and the action of the secreting and inhaling organs. Secondly, it is a solvent of various elementary substances and therefore assists the stomach in digestion, though if taken in very large quantities it may have an opposite effect by diluting the gastric juice. Thirdly, it is a nutritive agent, that is, it assists in the formation of the solid parts of the body.—*London Lancet.*

